# Hurricane power outage burden lands unequally: satellite evidence across 30 Atlantic storms

**DOI:** 10.21203/rs.3.rs-9349878/v1

**Published:** 2026-05-29

**Authors:** Chris Chaeha Lim, Cristian Román-Palacios, Ian Estacio, Paloma Beamer, Arnab Ghosh, Kai Zhang, Robbie M. Parks, G. Brooke Anderson, Michelle L. Bell

**Affiliations:** 1 Mel and Enid Zuckerman College of Public Health, University of Arizona, Tucson, AZ; 2 College of Information Science, University of Arizona, Tucson, AZ; 3 Arizona Institute for Resilience, University of Arizona, Tucson, AZ; 4 Department of Medicine, Weill Cornell Medicine, Cornell University, New York, NY; 5 Department of Population and Community Health, The University of North Texas Health Science Center at Fort Worth, Fort Worth, TX; 6 Department of Environmental Health Sciences, Columbia University Mailman School of Public Health, New York, NY; 7 Department of Environmental and Radiological Health Sciences, Colorado State University, Fort Collins, CO; 8 School of the Environment, Yale University, New Haven, CT

## Abstract

Hurricane-related outages affect millions, yet who loses power, how severely, and for how long remains poorly measured. Here we use satellite nighttime radiance to measure outage burden across 30 Atlantic hurricanes (2012–2024) and 156,032 tract-hurricane observations in 18 states. Decomposing outage burden into occurrence, severity and recovery, we find that occurrence disparities were the most robust: minority-status vulnerability was associated with 3.47 percentage points higher outage probability per 10-percentile-point increase. Severity disparities were positive and stronger among tracts that lost power, but attenuated after land-cover adjustment, indicating dependence on built-environment characteristics. Housing and transportation vulnerability was associated with slower recovery. Outages co-occurred with dangerous heat more often in high-minority tracts. Satellite monitoring can provide a utility-independent framework for tracking whether grid resilience investments reduce outage disparities.

Electricity is a core climate-adaptation resource, yet grid resilience is unevenly distributed. Atlantic hurricanes are intensifying under climate change and producing repeated major U.S. landfalls^1–3^. Power loss disables medical equipment, interrupts cooling during dangerous heat, and severs emergency communications, with documented increases in hospitalisation and mortality during and after tropical cyclone exposure^4–7^. Hurricane Irma caused the largest hurricane-related outage in Florida’s history^8^, and major landfalls have continued annually since. Yet aggregate outage figures obscure the key distributional questions: which communities lose power, how severely, and for how long. These questions have growing policy salience: the Infrastructure Investment and Jobs Act (2021), which remains in effect, channelled billions toward grid resilience, and the Justice40 initiative directed 40% of federal benefits to disadvantaged communities^9,10^. Although the federal Justice40 mandate was rescinded in January 2025^11^, several states, including Florida, California and Illinois, now require utilities to incorporate equity into resilience planning^12^, sustaining the need for standardised monitoring.

Whether socially patterned outage burdens reflect grid inequities, hazard geography or broader built-environment differences has been difficult to assess. Utility outage data are incomplete, unstandardised and not designed for equity monitoring: utilities report aggregate customer counts rather than demographic breakdowns, and although these could in principle be linked to census demographics at the ZIP code or county level, such linkages are rarely performed systematically^13–15^. Most prior studies examined single hurricanes^16–18^, limiting inference about recurring disparity patterns versus storm-specific geography. A recent multistate analysis using utility data found racial disparities in outage duration but could not disentangle infrastructure vulnerability from restoration prioritisation^19^. Evidence of differential investment in minority neighbourhoods across water systems, transit and electrical distribution networks^20–22^ suggests that grid inequities may be pervasive, but without standardised monitoring, regulators cannot determine whether investments are reducing disparities. That accountability gap becomes more urgent as climate change intensifies hurricane rainfall.

We use satellite-based nighttime light monitoring to address this gap. The Visible Infrared Imaging Radiometer Suite (VIIRS) provides daily nighttime radiance at 500-metre resolution, enabling tract-level measurement of radiance loss from outages independent of utility self-reporting^23–25^ (Figure 1). We analyse VIIRS radiance across 30 Atlantic hurricanes (2012–2024), covering 156,032 tract-hurricane observations in 18 states after applying the complete-case model criteria. We use the CDC Social Vulnerability Index (SVI)^26,27^ to decompose outage burden into three stages: which neighbourhoods lose power, how severe radiance deficits become and how quickly service-related nighttime activity recovers. This decomposition is useful because the three stages likely reflect different infrastructure processes and policy levers, including prevention of outage occurrence, limitation of outage severity, and restoration of service once outages occur. Because satellite radiance is available for most communities after most storms (subject to cloud-cover limitations), this approach can serve as a utility-independent accountability tool when conventional outage reporting cannot support equity oversight.

## RESULTS

### Summary Statistics.

**Figure 2:**
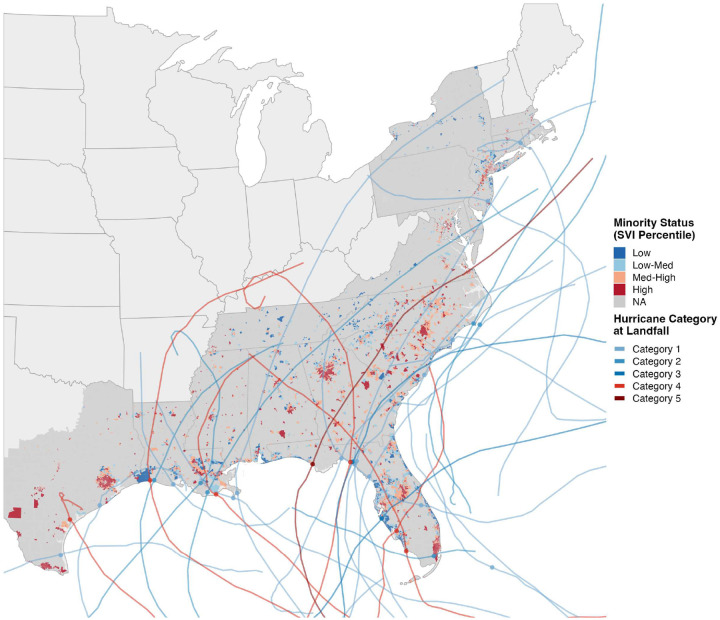
Geographic extent and hurricane tracks. All census tracts within hurricane wind radii across 30 Atlantic hurricanes (2012–2024) and 18 states. Grey tracts fell within the R34 wind-radius exposure definition but did not experience detectable outages; coloured tracts experienced outages and are shaded by minority status (SVI Theme 3 percentile; blue, low-minority; red, high-minority). The map shows 39,467 unique census tracts exposed across all storms, of which 19,685 experienced at least one detectable outage. Hurricane tracks are coloured by Saffir-Simpson category at landfall (light blue, Category 1–2; dark blue, Category 3; red, Category 4–5). Dots indicate landfall locations.

Across 30 continental U.S. hurricanes in 18 states (2012–2024), the R34 exposure sample contained 194,309 tract-hurricane observations, of which 156,032 had complete data for the three-stage models, representing approximately 617 million person-storm exposure events (Table 1; Figure 2). Of these analytic observations, 50,441 (32.3%) experienced detectable outages (radiance deficit >1 nW/cm^2^/sr) and were included in recovery analyses. Mean 3-day radiance deficit among affected tracts was 8.3 nW/cm^2^/sr (s.d.: 12.6), and 30-day recovery averaged 77%. The sample spans Category 1 systems to major hurricanes, including major Gulf and Southeast landfalls such as Harvey (2017), Ida (2021) and Helene (2024).

### A three-stage pattern of disparity.

Disparities in outage burden emerged at three stages: occurrence, severity and recovery (Extended Data Table 1). All main-text coefficients are reported per 10-percentile-point (10pp) increase in SVI vulnerability unless otherwise noted. Across stages, occurrence showed the clearest residual disparity pattern after adjustment, severity was the most dependent on geographic and land-cover controls, and recovery was intermediate in robustness.

In the mutually adjusted four-theme model, Theme 3 (Minority Status/Language) showed the strongest association with whether tracts lost power and with unconditional outage severity, whereas Theme 4 (Housing Type/Transportation), which captures mobile homes, crowded housing and limited vehicle access, was the only theme significantly associated across all three stages. Theme 1 (Socioeconomic Status) showed a negative association with outage probability (−0.84 pp per 10pp; *P* < 0.001), and Theme 2 (Household Composition/Disability) showed null or suppression-driven patterns under mutual adjustment (see Methods; Extended Data Tables 3 and 4).

### Stage 1: Outage probability.

**Figure 3:**
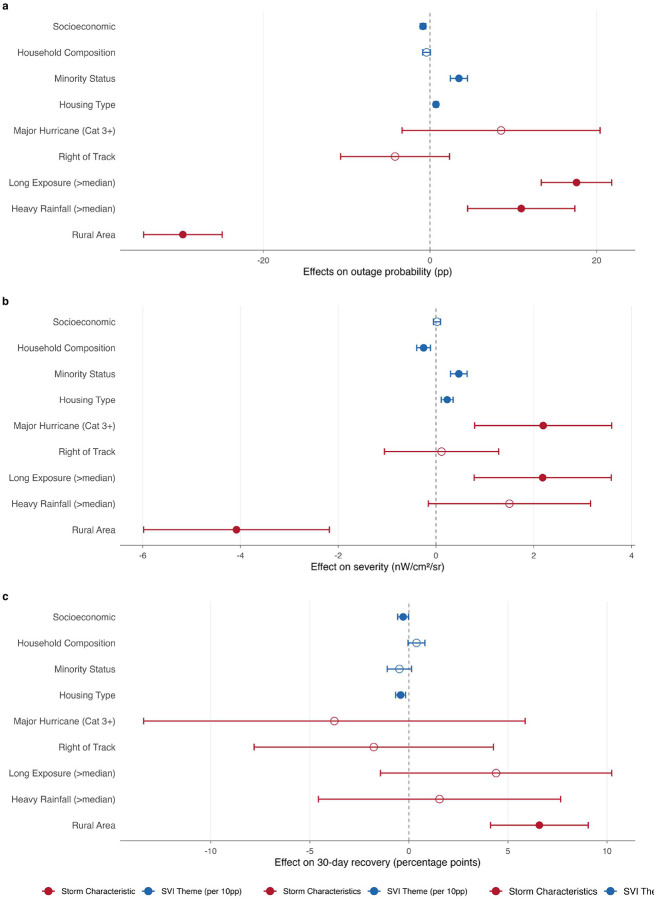
Social vulnerability and storm characteristic effects across three outage stages. SVI theme coefficients (blue) from the four-theme joint model; storm characteristics (red) from separate pooled models (severity and recovery only). All per 10-percentile-point SVI increase with two-way clustered SEs (state and hurricane). a, Outage probability (pp). b, Outage severity (nW per cm squared per sr). c, 30-day recovery (pp; negative equals slower). Filled circles, P less than 0.05; open circles, not significant.

Theme 3 showed the largest occurrence gap (Figure 3, panel a): each 10-percentile-point increase in SVI vulnerability was associated with 3.47 pp higher probability of any detectable outage (95% confidence interval (CI): 2.44 to 4.50; *P* < 0.001). This translates to roughly a 17 pp difference between the 25th and 75th SVI percentiles. Within Theme 3, percent minority was the more consistent driver of the occurrence association, with limited English proficiency showing a weaker and less stable pattern (Extended Data Table 7). Theme 4 was also associated with higher outage probability (+0.72 pp per 10pp; *P* < 0.001), reflecting greater vulnerability in tracts with mobile homes, crowded housing and limited vehicle access.

### Stage 2: Outage severity.

In the unconditional model (all exposed tracts, including zeros for tracts without detectable outages), Theme 3 was associated with +0.47 nW/cm^2^/sr greater radiance deficit per 10pp (*P* < 0.001) and Theme 4 with +0.23 nW/cm^2^/sr (*P* < 0.001) (Figure 3, panel b). Conditional on any outage, both associations were substantially larger and highly significant (Theme 3: +0.77; Theme 4: +0.56; both *P* < 0.001), indicating that severity disparities are concentrated among tracts that lost power. Across storm-specific single-theme severity models, the Theme 3 coefficient was positive in 29 of 30 estimable hurricanes and statistically significant in 28 of them (Figure 6).

### Stage 3: Recovery pace.

Among tracts that lost power, Theme 4 was associated with a 0.42 pp lower 30-day recovery rate per 10pp (95% CI: −0.67 to −0.16; *P* = 0.003). Theme 3 showed a directionally similar but weaker association (−0.48 pp per 10pp; *P* = 0.12). Because recovery is defined only for tracts that experienced outages, recovery models may be susceptible to collider bias when the determinants of outage occurrence also influence restoration speed (see Methods). In the 2017–2024 subsample, the Theme 3 recovery association remained negative but imprecise, whereas the Theme 4 association remained significant (Extended Data Table 5, Panel C).

### Rainfall amplification and spatial context.

**Figure 4:**
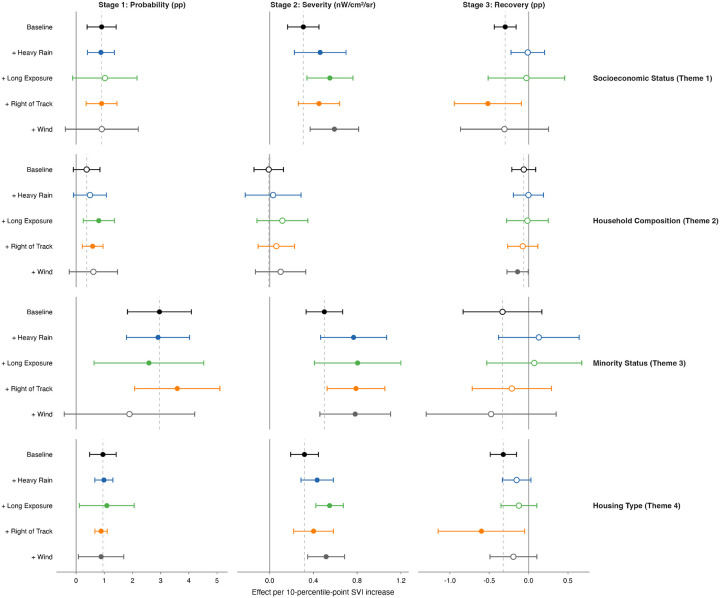
Storm characteristics modify social vulnerability associations across the three-stage cascade. Each point shows the SVI theme association under a given storm condition: baseline (no modifier), heavy rainfall (above sample median precipitation), long exposure (above 75th percentile duration), right of track, and wind speed (standardised within hurricane). Estimates are from single-theme interaction models with hurricane fixed effects and two-way clustered standard errors (state + hurricane), per 10-percentile-point SVI increase. Columns: Stage 1, outage probability (pp); Stage 2, severity (nW/cm^2^/sr); Stage 3, recovery (pp; negative = slower). a, Socioeconomic Status (Theme 1). b, Household Composition/Disability (Theme 2). c, Minority Status/Language (Theme 3). d, Housing Type/Transportation (Theme 4). Filled circles, *P* < 0.05; open circles, not significant. Error bars, 95% CIs.

**Figure 5:**
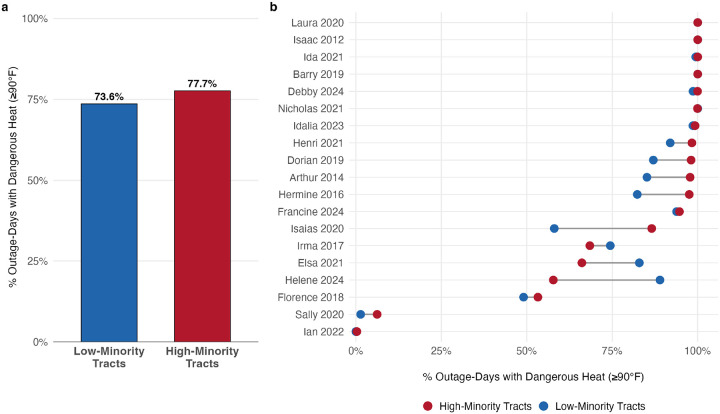
Compound heat-outage risk by community minority status. **a**, Among tracts experiencing outages during warm-season hurricanes (June–September), 77.7% of outage-tract observations in high-minority tracts co-occurred with dangerous heat (daily maximum ≥90 °F, PRISM 4-km daily data) versus 73.6% in low-minority tracts, partly reflecting geographic sorting of minority populations into warmer places. **b**, Hurricane-specific heat co-occurrence for warm-season storms with sufficient data (≥50 outage tracts per group). Each row shows the percentage of outage-tract observations with dangerous heat in high-minority (red) and low-minority (blue) tracts. The gap between dots represents the differential heat burden. Storms are sorted by the high-minority co-occurrence rate.

Storm characteristics modified the severity association, with rainfall showing a clear amplification (Figure 4; Extended Data Table 2). Heavy rainfall (above the sample median precipitation) was associated with a 0.49 nW/cm^2^/sr larger Theme 3 severity gap per 10pp (*P* < 0.05). Longer exposure duration, right-of-track position, and higher within-hurricane wind speed also yielded positive Theme 3 severity interactions in exploratory models (Extended Data Table 2). In stratified diagnostics, Theme 3 severity was positive in both rural and urban tracts, and the rural-urban interaction was not statistically distinguishable (*P* = 0.20). Among tracts experiencing outages during warm-season hurricanes, dangerous heat co-occurred more often in high-minority than low-minority tracts (Figure 5).

### Cross-storm consistency.

**Figure 6:**
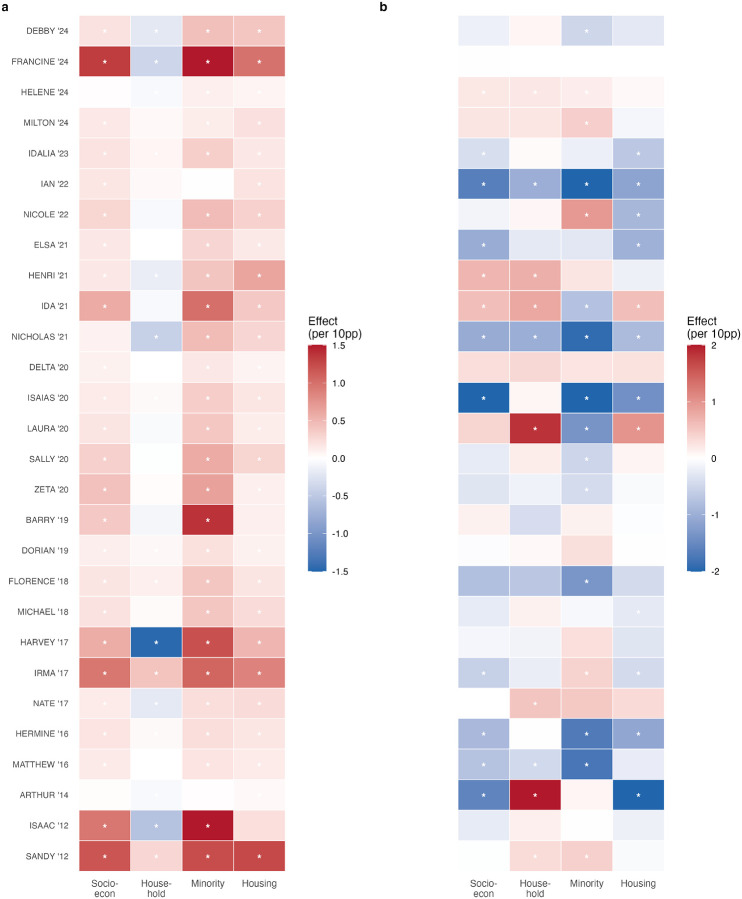
Hurricane-specific social vulnerability effects on outage severity and recovery. **a**, Effects on outage severity (3-day radiance deficit). **b**, Effects on 30-day recovery rate. Each cell shows the coefficient for a specific hurricane-theme combination from hurricane-specific single-theme models (per 10-percentile-point increase in SVI theme, with wind speed and distance controls; heteroskedasticity-robust standard errors). Red indicates higher vulnerability worsens outcomes; blue indicates improvement. Asterisks indicate *P* < 0.05. Hurricanes are ordered by year.

**Figure 7:**
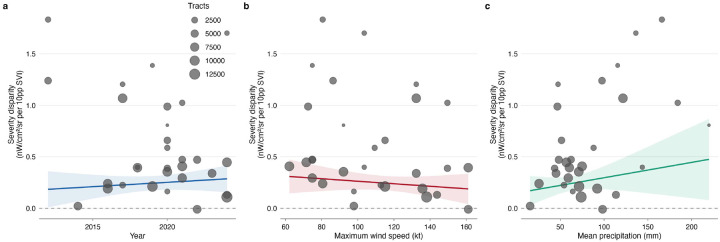
Between-hurricane patterns in minority severity disparities. **a**, Hurricane-specific minority severity disparity (Theme 3 coefficient, nW/cm^2^/sr per 10pp SVI) plotted against year, with inverse-variance weighted regression line and 95% confidence interval. In the meta-regression adjusting for maximum wind speed and mean precipitation, the year coefficient is close to zero and not statistically distinguishable from no trend (*P* = 0.55; model R^2^ = 0.15). **b**, Severity disparity versus maximum wind speed (kt). c, Severity disparity versus mean precipitation (mm). Between-hurricane variation by wind and precipitation is suggestive but imprecise in the meta-regression, even though within-hurricane rainfall amplification remains positive in the interaction models. Point sizes are proportional to the number of tracts. Hurricane names labelled for identification.

The Theme 3 severity coefficient was positive in 29 of 30 estimable hurricanes and significant in 28, despite substantial heterogeneity across storms (Figure 6). Stratification by hurricane category showed positive Theme 3 severity associations in both Category 1–2 and Category 3+ storms, with larger magnitudes for major hurricanes (Extended Data Table 6, Panel C). A broader inland sensitivity sample for six storms also remained positive (+0.50 nW/cm^2^/sr per 10pp; *P* = 0.005), suggesting that disparity is not limited to the immediate coastal wind field. Exploratory between-hurricane temporal trends were weak (Figure 7).

### Stage-specific robustness to geographic and land-cover adjustment.

The preceding associations could reflect confounding by hazard geography or the urbanisation gradient (built-environment differences correlated with both demographics and infrastructure quality). We tested two independent robustness approaches: within-county × hurricane fixed effects and tract-level land-cover controls. Theme 3 unconditional severity attenuated from +0.47 in the base specification to +0.21 with tree canopy alone and to approximately zero after adding impervious surface, whereas the within-county estimate remained positive (+0.14; *P* = 0.002). Theme 4 severity was more stable, remaining positive under both within-county fixed effects (+0.25; *P* < 0.001) and the combined land-cover specification (+0.11; *P* < 0.05). Probability disparities were more robust than severity disparities: Theme 3 occurrence attenuated from +3.50 to +0.70 pp under the combined land-cover specification but remained significant (*P* < 0.001). Recovery evidence under the most stringent specifications was concentrated in Theme 4, not Theme 3 (Extended Data Table 6).

### Disparities persist within utility service territories.

As an additional test, we linked tracts to public electric utility service-territory polygons and re-estimated the three stages on the matched sample. The utility assignment covered 35,751 of 39,467 unique tracts (90.6%), spanning 327 utilities and 96.6% of Stage 1–2 observations. Under the strongest utility × hurricane fixed-effects specification, Theme 3 remained associated with higher outage probability (+3.39 pp per 10pp; *P* < 0.001) and greater severity (+0.46 nW/cm^2^/sr; *P* < 0.001), while the recovery coefficient remained negative but imprecise (−0.57 pp; *P* = 0.056). Theme 4 recovery remained significant (−0.42 pp; *P* < 0.01) (Extended Data Table 5, Panel D). This pattern is consistent with, but not diagnostic of, neighbourhood-level differences contributing to disparity beyond utility-wide composition.

## DISCUSSION

Across 30 hurricanes, minority and housing-vulnerable communities were more likely to lose power, experienced deeper outages when power was lost, and in the case of housing vulnerability, recovered more slowly. These disparities are not confined to restoration: they begin at outage occurrence, which showed the most robust pattern after adjustment. By contrast, severity disparities were common but more sensitive to spatial and built-environment adjustment, whereas recovery disparities were weaker overall but remained directionally consistent and persisted in several stringent specifications.

Severity tells a more nuanced story. Across the full exposure zone, the minority-status severity association is positive but sensitive to how the sample is defined and to built-environment adjustment. Among tracts that actually lost power, the association is stronger and highly significant (+0.77 nW/cm^2^/sr per 10pp; *P* < 0.001). This pattern suggests that when minority communities lose power, they lose more of it, but that this disparity is closely tied to the urbanisation gradient and to spatial patterns in infrastructure investment^28,29^. Because the severity metric captures absolute radiance loss rather than direct household service status, it reflects both outage burden and pre-existing development intensity. Occurrence, which uses a binary threshold, is less susceptible to this confound. Whether land cover confounds or mediates the severity association cannot be resolved with observational data. We report both unconditional and conditional specifications to bracket these interpretations (see Methods). Themes 3 and 4 retain independent associations despite moderate correlation (*r* = 0.33–0.56; VIF < 2.1). The contribution is a stage-specific framework for identifying where disparities remain detectable after increasingly strict adjustment, rather than an attempt to isolate a single feeder-level mechanism.

Three patterns in the data are consistent with an important role for infrastructure and built-environment characteristics. First, rainfall amplified the minority-severity association, whereas wind interactions were weak, pointing to pathways linked to flooding, vegetation or urban form rather than to wind damage alone^30,31^. Second, occurrence disparities were concentrated in tracts with lower baseline radiance (a proxy for nighttime development intensity) rather than varying systematically with storm intensity. Third, within-county comparisons retained occurrence and recovery associations while sharply attenuating severity, suggesting that which tracts lose power and how quickly service returns are not fully explained by county-level hazard geography alone. A utility-territory sensitivity analysis using public service-boundary polygons yielded a similar pattern under utility × hurricane fixed effects. Because those boundaries are imperfect and overlapping in some places, this analysis should be interpreted as supportive rather than definitive.

Most existing equity discussions in grid resilience focus on restoration time^17,19^. If disparities are already evident at outage occurrence, then an equity strategy focused only on restoration is incomplete. The three-stage framework helps distinguish where different interventions may matter most. Prevention-oriented investments, including system hardening, undergrounding and vegetation management, are most relevant to outage occurrence. Land-use and infrastructure investment may shape how severe disruption becomes once outages occur. Restoration protocols, dispatch priorities and operational decision rules are most relevant to recovery^17^. The persistence of occurrence and severity disparities within utility territories, together with the more stable Theme 4 recovery association, is consistent with, but not diagnostic of, neighbourhood-level differences in network design, maintenance or restoration conditions contributing alongside utility-wide composition. Public utility commissions could use satellite-derived equity metrics as one accountability layer within resilience planning, especially where utility reporting is incomplete, inconsistent or not designed for distributive oversight^32^.

The higher rate of heat-outage co-occurrence in high-minority tracts partly reflects geographic sorting into warmer places, but the gap persists within states (6.7 pp; *P* < 0.001) and within state-by-hurricane cells (4.6 pp; *P* < 0.001), indicating that within-storm differences also contribute. Regardless of mechanism, high-minority tracts more often experienced simultaneous power loss and dangerous heat, a particularly hazardous combination because loss of air conditioning during extreme heat is a well-established pathway to heat-related illness and mortality^33,34^, and tropical cyclone exposure is itself associated with excess mortality from cardiovascular, respiratory and neuropsychiatric causes^35,36^. These patterns connect to broader work on energy insecurity^37,38^, suggesting that hurricane-related outages represent an acute, event-driven dimension of a chronic disparity in energy access.

Ganz and colleagues^15^ found that socioeconomically vulnerable communities experienced disproportionate outage impacts from severe weather using county-level utility data, though the coarse spatial resolution limited their ability to distinguish neighbourhood-level mechanisms. A recent three-state study of weather-related outages (not limited to hurricanes) attributed much of the racial disparity to restoration processes^19^. Using a measurement system independent of utility self-reporting, we instead find that disparities are detectable before restoration begins and recur across a much larger set of storms. Where utility data are strongest for measuring customer restoration time, satellite radiance captures a tract-level proxy for the broader outage lifecycle, including the occurrence gap that conventional utility reporting cannot easily assess. The recurrence of these patterns across 30 hurricanes suggests that outage inequity may reflect structural features of energy systems rather than isolated storm-specific anomalies. Because VIIRS measures ambient outdoor radiance rather than customer-level service status, it should be viewed as a complement to, rather than a substitute for, granular utility data^13^.

Several limitations should be noted. First, VIIRS measures outdoor nighttime radiance, not household electricity service. Because commercial lighting and public illumination contribute to tract-level radiance, satellite-detected outage patterns may not map perfectly onto residential experience, and all results should be interpreted at the tract level. Second, the SVI themes are area-level screening constructs that aggregate heterogeneous census variables; they are informative about neighbourhood characteristics but do not identify which individuals within a tract bear the greatest burden. Component decomposition suggests that percent minority was the more consistent Theme 3 contributor whereas limited English proficiency showed less stable evidence across specifications; finer-grained analysis of which populations within minority-designated tracts bear the greatest outage burden is beyond the scope of these ecological data. Third, we cannot observe evacuation, backup generation or distribution-system design. Differential access to generators, which likely varies with income and housing type, may reduce observed radiance deficits in some tracts even when grid outages occur. More broadly, the rapid expansion of distributed energy resources (rooftop solar and battery storage), which is also income- and race-correlated^22^, may increasingly affect satellite-detected outage patterns in ways this analysis cannot capture. Fourth, post-disaster displacement may alter the effective population at risk during recovery, and our recovery outcome is right-censored at 30 days. Finally, recovery models are conditional on outage occurrence and may therefore be susceptible to collider bias when the determinants of outage occurrence also influence restoration speed. Because cloud-related missingness in the satellite data was both socially and meteorologically patterned (concentrated in brighter, rainier tracts), the direction of any resulting bias is not assured; our days-3-to-7 and subsample sensitivity analyses reduce but do not eliminate this concern.

Additional limitations relate to measurement and temporal alignment. The utility-territory sensitivity analysis relies on public boundary polygons and overlap rules rather than utility-reported feeder maps. Although utility assignment covered most tracts and supported the main Theme 3 probability and severity pattern, residual boundary error means that this exercise cannot fully separate neighbourhood-level inequity from measurement error in service territories. Applying 2020 SVI to storms spanning 2012–2024 also introduces temporal mismatch, although the 2017–2024 subsample produced broadly similar occurrence and severity estimates and retained the negative Theme 3 recovery direction (Extended Data Table 5, Panel C). Census tract geography further excludes Puerto Rico, where hurricane-related outages have been especially severe and prolonged^39^.

Given that Hurricanes Maria and Fiona represent arguably the most consequential outage equity cases in this study period, the exclusion limits generalizability precisely where it may matter most. Future work linking satellite-based outage measures to higher-resolution utility infrastructure data could help distinguish more clearly among grid design, land cover and restoration governance.

The three stages tell different stories. Occurrence disparities survived every robustness check we applied. Severity disparities were real among tracts that lost power but were shaped by urban form and land cover. Recovery disparities were most consistent for housing and transportation vulnerability. Each stage points to a different policy lever, and each responds differently to geographic adjustment. With hurricane precipitation projected to increase by 10–15% under climate change^40^, and with rainfall amplifying the severity disparity in our data, these patterns are unlikely to improve without targeted investment. VIIRS nighttime radiance cannot replace utility records, but it is available within days of each landfall and covers every affected community uniformly. That makes it a practical tool for regulators who need to evaluate whether resilience spending is reaching the communities that bear the greatest outage burden.

## METHODS

### Study population and data sources:

We identified Atlantic hurricanes making US continental landfall between 2012 and 2024 from the International Best Track Archive for Climate Stewardship (IBTrACS)^41^. Of the tropical cyclones affecting US territory during this period, we excluded: (1) Beryl (2024), for which VIIRS Black Marble data were unavailable at study completion; (2) Maria (2017) and Fiona (2022), which primarily impacted Puerto Rico and use different census geography incompatible with the CDC SVI framework; (3) Fred (2021), which never reached hurricane intensity. The final sample comprised 30 hurricanes with VIIRS data extraction completed. For 2024 storms (Debby, Francine, Helene, Milton), we used IBTrACS preliminary best-track data and near-real-time meteorological products, both of which were available at study completion; final reprocessed versions may differ slightly. The impact date used for each hurricane reflects the analysis anchor (typically landfall date or the following calendar day in UTC), not necessarily the first NHC advisory. For storms with multiple landfalls (for example, Eta 2020), the anchor corresponds to the primary impact on the study area.

### Exposure definition:

Census tracts were included if their centroids fell within the National Hurricane Center’s wind radii buffers (R34, R50, R64) extracted from IBTrACS best-track data. For modelling, we defined a detectable outage as a 3-day radiance deficit >1 nW/cm^2^/sr. For descriptive population-burden estimates, we used a stricter >2 nW/cm^2^/sr threshold to reduce noise from minor radiance fluctuations. As a secondary definition, we also generated peak sustained wind at each tract centroid using the Willoughby parametric wind profile model^42^, defining tropical cyclone-force exposure as tracts where modelled wind reached or exceeded 34 knots (17.5 m/s). This provides an alternative exposure definition for sensitivity analysis.

### Sample derivation:

The initial R34 exposure dataset included 194,309 tract-hurricane observations (one census tract observed for one hurricane). We excluded observations with missing baseline or 3-day post-storm radiance (36,109; 18.6%), missing or invalid SVI theme scores (2,810; 1.4%), or missing wind speed or distance-to-track values (154; <0.1%); these categories partially overlap. Wind speed gaps were addressed using a time-windowed nearest-track-point approach (±2 days) with radial decay correction, reducing missing wind-speed values to a negligible remainder. The resulting analytic sample comprised 156,032 tract-hurricane observations across 30 hurricanes and 18 states, of which 50,441 (32.3%) experienced detectable outages (radiance deficit >1 nW/cm^2^/sr) and were included in recovery analyses.

Nighttime radiance data were obtained from NASA’s Black Marble VIIRS Day/Night Band daily product (VNP46A2) via Google Earth Engine^43,44^. We used the cloud-masked DNB_BRDF_Corrected_NTL radiance band at 500-m resolution and extracted tract-level mean radiance at baseline (15–30 days before landfall) and at 3, 7, 14 and 30 days post-landfall.

### Meteorological data:

Daily maximum temperature was extracted from the PRISM gridded dataset (Parameter-elevation Regressions on Independent Slopes Model; ~4-km resolution; Oregon State University PRISM Climate Group)^45^ via Google Earth Engine. Dangerous heat was defined as daily maximum ≥90 °F, an absolute threshold chosen for interpretability in outage-health contexts. Precipitation was extracted from ERA5-Land reanalysis data (~9-km resolution; European Centre for Medium-Range Weather Forecasts, ECMWF) via Google Earth Engine; we calculated total accumulation during the 4-day landfall window. Because gridded reanalysis can smooth the most extreme hurricane rainfall, this modifier should be interpreted as a standardised storm-context measure rather than a gauge-equivalent estimate of peak local rainfall.

### Tree canopy and impervious surface data:

Tract-level percent tree canopy cover was obtained from the MODIS Vegetation Continuous Fields product (MOD44B, Collection 6), which provides annual estimates of percent tree cover at 250-m resolution; we used the 2020 annual composite. Tract-level percent impervious surface cover was obtained from the NLCD 2021 Impervious Surface product (30-m resolution). For both variables, we calculated the mean percentage within each census tract boundary via Google Earth Engine.

### Social Vulnerability:

Social vulnerability measures were obtained from the CDC/Agency for Toxic Substances and Disease Registry (ATSDR) Social Vulnerability Index 2020^26,27^. The SVI ranks every U.S. census tract on 16 social factors grouped into four themes. Theme 1 (Socioeconomic Status) includes below 150% poverty, unemployment, housing cost burden, no high-school diploma and no health insurance. Theme 2 (Household Composition/Disability) includes aged 65+, aged 17 and under, civilian disability and single-parent households. Theme 3 (Minority Status/Language) includes minority race/ethnicity percentage and limited English proficiency. Theme 4 (Housing Type/Transportation) includes multi-unit structures, mobile homes, crowding, no vehicle and group quarters. Each theme is reported as a national percentile ranking (0–100); in our analytic files these are stored on a 0–1 scale (percentile/100), where higher values indicate greater vulnerability. SVI is an area-level screening index rather than an individual-level risk score, so we use it to characterise tract context rather than to infer that every resident in a tract has the same vulnerability profile. All four themes were estimated in parallel. Prior literature documents racial disparities in disaster impacts, motivating particular attention to Theme 3; for brevity we refer to this composite as minority status and note it includes limited English proficiency. We applied the 2020 SVI vintage to hurricanes spanning 2012–2024, introducing potential temporal mismatch (see Temporal Mismatch Sensitivity below).

### Outcome measures:

Outage severity was measured as the absolute radiance deficit at 3 days post-landfall. The 3-day metric uses the day-2 to day-4 post-landfall composite, which balances the need to capture widespread disruption after storm passage against the higher cloud contamination and coastal-inland timing mismatch present immediately at landfall; we also report a sensitivity analysis using the maximum deficit over days 3–7.


$${\text{Deficit}}_{3d}=\text{max}\left(0,{\text{Radiance}}_{\text{baseline}}-{\text{Radiance}}_{\text{post}3\text{d}}\right)$$

Recovery rate was measured as the proportion of initial deficit recovered by 30 days:

$${\text{Recovery}}_{30}=\frac{{\text{Deficit}}_{3d}-{\text{Deficit}}_{30d}}{{\text{Deficit}}_{3d}}$$

### Prior hurricane exposure:

Prior hurricane exposure was defined as the count of previous hurricanes in our study period (2012–2024) for which each census tract experienced a detectable outage before the index hurricane. This variable is therefore left-censored for early study years and should be interpreted as prior exposure within the observation window rather than complete lifetime hurricane history.

### Statistical analysis:

We estimated a three-stage decomposition framework to avoid selection bias from conditioning on outage occurrence, which would inflate severity estimates by comparing only tracts that lost power.

### Stage 1: outage probability:

We estimated a linear probability model for any detectable outage (deficit >1 nW/cm^2^/sr) among all 156,032 exposed tract-hurricane observations:

$$P\left({\text{Outage}}_{it}=1\right)=\beta \cdot \text{SVI}+\gamma \cdot X+\alpha_{h}+\varepsilon$$

### Stage 2: outage severity:

We estimated outage severity across all exposed tracts, including zeros for tracts without detectable outages. This unconditional estimate captures the total severity burden and is not subject to selection bias:

$${\text{Deficit}}_{3d}=\beta \cdot \text{SVI}+\gamma \cdot X+\alpha_{h}+\varepsilon \;\left(\text{all exposed tracts}\right)$$

### Stage 3: recovery pace:

Recovery can only be measured for tracts that experienced outages, making conditional analysis appropriate but introducing potential collider bias if factors that determine outage occurrence also influence recovery speed. We estimated recovery among the 50,441 tracts with detectable outages, controlling for initial deficit magnitude:

$${\text{Recovery}}_{30}=\beta \cdot \text{SVI}+\delta \cdot \text{log}\left(\text{InitialDeficit}\right)+\gamma \cdot X+\alpha_{h}+\varepsilon$$

Recovery is bounded on [0, 1], and the linear model can in principle generate fitted values outside this range. We retain the linear specification for interpretability and consistency across stages; a fractional logit produced the same direction and inference pattern.

In all models, SVI themes were entered on the 0–1 scale (where 1 = highest vulnerability, equivalent to the 100th percentile); all coefficients are reported per 10-percentile-point (0.10-unit) increase throughout the manuscript. X includes control variables (wind speed, distance to track) and **α**_h_ are hurricane fixed effects. Standard errors were two-way clustered by state and hurricane using the fixest package^46^, accounting for both cross-hurricane correlation within states (tracts experiencing multiple hurricanes) and within-hurricane spatial correlation (adjacent tracts affected by the same storm). Confidence intervals and *P*-values use the *t*-distribution with min(*G*_1_, *G*_2_) − 1 = 17 degrees of freedom. Main text reports four-theme joint estimates unless otherwise noted. Interaction models and hurricane-specific estimates use single-theme specifications, which yield larger Theme 3 severity coefficients than the mutually adjusted model because correlated themes are not simultaneously included.

### Effect modification:

The core adjustment set (all models) includes wind speed, distance to track, and hurricane fixed effects. Effect modification models are estimated separately from the main three-stage models and include one SVI theme × storm characteristic interaction at a time (Figure 4). The narrative highlights Theme 4 (significant at all three stages) and Theme 3 (significant at Stages 1 and 2, with a directionally consistent but marginally non-significant Stage 3 association); full results for all four themes are shown in Figure 4. We used four pre-specified storm modifiers: heavy rainfall (precipitation above the sample median during the 4-day landfall window), long exposure (exposure duration at or above the 75th percentile), right-of-track position (dangerous semicircle indicator), and wind speed standardised within hurricane. Because we test four modifiers across four themes and three stages, the interaction results should be interpreted as exploratory; we highlight only the heavy rainfall × Theme 3 severity interaction in the main text and present remaining interactions in Figure 4 without correction for multiplicity.

### Infrastructure and geographic context:

Rural-urban classification was based on Census Bureau urban-rural definitions at the tract level. Infrastructure age was proxied by median year of housing construction from the American Community Survey 5-year estimates. We estimated interaction models (SVI theme × rural indicator; SVI theme × housing age decile) to test whether infrastructure and geographic context modified vulnerability associations, focusing on Theme 3 in the narrative.

### SVI component decomposition:

To identify which aspects of social vulnerability drive the composite associations, we estimated separate models replacing SVI themes with constituent census variables (for example, percent minority and limited English proficiency from Theme 3; no vehicle, mobile homes and housing cost burden from Theme 4), each with hurricane fixed effects and two-way clustered standard errors.

### Meta-regression:

To test whether disparities vary with storm intensity or over time, we estimated inverse-variance weighted meta-regression models using hurricane-specific severity coefficients as the outcome and year, maximum wind speed and mean precipitation as predictors. We focus on Theme 3 coefficients given the policy relevance of racial disparities in disaster outcomes.

### Heat co-occurrence analysis:

Among tracts experiencing outages during warm-season hurricanes (June–September), we calculated the percentage of tract-hurricane observations where a detectable outage co-occurred with dangerous heat (daily maximum temperature ≥90 °F from PRISM daily gridded data). We stratified tracts by median SVI Theme 3 score into high-minority and low-minority groups, given the documented association between race/ethnicity and heat-related mortality, and compared co-occurrence rates. We additionally estimated linear probability models for heat co-occurrence with state fixed effects and state × hurricane fixed effects to assess within-state and within-storm gaps. For hurricane-specific comparisons, we required ≥50 outage tracts per group.

### SVI Theme 2 interpretation:

The protective association observed for SVI Theme 2 (Household Composition/Disability) in joint models requires cautious interpretation. In single-theme models, Theme 2 showed a non-significant positive association with outage severity; the negative association emerged only under mutual adjustment with correlated themes (pairwise r = 0.33–0.56). This sign flip is a classical suppression artefact. We thus do not interpret the Theme 2 coefficient as a causal protective effect.

### Sensitivity analyses:

Wild cluster bootstrap inference for the severity model was used as a precision check for the main SVI themes and selected component and storm-characteristic models (Extended Data Table 4). We did not bootstrap the recovery model because the conditional sample yields variable effective sizes across bootstrap draws when resampling hurricane clusters, reducing convergence stability. Adding population density as a control changed the Theme 3 severity estimate by 27%, indicating that development intensity explains part, but not all, of the radiance-deficit association. Variance inflation factors were low (range: 1.04–2.05). Moran’s *I* test on a 20,000-tract sample of severity-model residuals showed significant spatial autocorrelation (*I* = 0.25; *P* < 0.001), suggesting our two-way clustering may underestimate local spatial dependence. Conley spatial heteroskedasticity and autocorrelation consistent (HAC) standard errors with 50-km and 100-km bandwidths were smaller than the two-way clustered standard errors for the key severity coefficients, so we retained two-way clustering as the more conservative approach. An inland sensitivity analysis using a broader storm-impact definition for six storms also yielded a positive Theme 3 severity association (+0.50 nW/cm^2^/sr per 10pp; *P* = 0.005).

### Baseline radiance and outcome specification:

Because absolute radiance deficit can scale mechanically with baseline brightness, we estimated two alternative specifications: (1) relative deficit (deficit divided by baseline radiance) as the outcome, and (2) the absolute deficit model with baseline radiance added as a covariate. The relative-deficit specification attenuated Theme 3 severity to near zero, whereas adding baseline radiance as a covariate reversed the Theme 3 sign, underscoring that baseline brightness absorbs substantial built-environment variation (Extended Data Table 5, Panel A). However, baseline radiance is endogenous to the infrastructure conditions under study, so we treat the within-county × hurricane fixed-effects model as the preferred robustness specification for separating infrastructure inequity from hazard geography. Stratification by baseline-radiance tertile showed a small positive Theme 3 severity estimate in low-baseline tracts and a larger positive estimate in high-baseline tracts, indicating that absolute radiance deficits behave differently across development contexts.

### Within-county robustness:

To address the concern that demographic composition is confounded with hazard geography (for example, minority communities sorted into flood-prone areas), we estimated models with county × hurricane fixed effects, comparing tracts within the same county during the same hurricane and thus under similar hazard conditions. Theme 3 remained significant at all three stages under within-county fixed effects, whereas Theme 4 remained significant for outage probability and severity and was weaker for recovery (*P* = 0.037). Within-county magnitudes were attenuated relative to the pooled model (for example, Theme 3 severity: 0.14 versus 0.47 nW/cm^2^/sr per 10pp in the four-theme joint model), consistent with between-county hazard geography contributing to pooled estimates (Extended Data Table 6, Panel B).

### Utility-territory sensitivity:

To assess whether between-utility differences in maintenance, capital investment or restoration protocols explained the tract-level associations, we linked analysis tracts to public utility service-territory polygons from the Homeland Infrastructure Foundation-Level Data layer and 2020 Census tract geometries. Tracts were assigned to the utility with the largest area overlap; for tracts without polygon overlap we used a point-on-surface nearest-territory rule within 20 km. This procedure assigned 35,751 of 39,467 unique tracts (90.6%) to 327 utilities, covering 150,670 of 156,032 Stage 1–2 observations and 49,451 of 50,441 recovery observations. We then re-estimated the three stages on the matched sample with hurricane fixed effects, utility fixed effects, and utility × hurricane fixed effects, clustering standard errors by utility and hurricane. Because utility assignment is derived from public boundaries and overlap rules rather than utility-reported feeder maps, we treat this as a sensitivity analysis rather than the primary specification (Extended Data Table 5, Panel D).

### Missingness analysis:

We characterised the 36,109 observations (18.6%) with missing post-storm radiance. Missing tracts had higher baseline radiance (29.2 versus 20.1 nW/cm^2^/sr) and higher precipitation (106.9 versus 74.8 mm), consistent with cloud contamination during rainy storms disproportionately affecting brighter, more developed tracts. Missingness was also associated with slightly higher Theme 3 scores (0.58 versus 0.54; *P* < 0.001), so we report complementary severity specifications, including the maximum deficit over days 3–7, to reduce sensitivity to single-day cloud loss (Extended Data Table 5, Panel B).

### Temporal mismatch sensitivity:

To assess sensitivity to applying 2020 SVI data to earlier hurricanes, we restricted the sample to 2017–2024 storms. Theme 3 probability (+3.44 pp per 10pp; *P* < 0.001) and Theme 3 severity (+0.47; *P* < 0.001) remained similar to the full-sample estimates, as did the Theme 4 severity and recovery associations. The Theme 3 recovery coefficient remained negative but imprecise, suggesting that the main occurrence and severity findings are not driven by temporal mismatch between SVI vintage and storm year (Extended Data Table 5, Panel C).

### Additional model specifications:

A two-part decomposition separating probability from conditional severity among outage tracts supported that Theme 3 (+0.77 nW/cm^2^/sr per 10pp; *P* < 0.001) and Theme 4 (+0.56; *P* < 0.001) associations are present within the outage subsample; the larger conditional estimates relative to the unconditional Stage 2 estimates reflect the expected inflation from restricting to tracts that lost power. A logit model for Stage 1 (outage probability) produced the same direction and significance pattern as the linear probability model (Theme 3 odds ratio (OR) = 1.21 per 10pp; *P* < 0.001; Theme 4 OR = 1.04; *P* < 0.001). Two-way clustering by hurricane and tract yielded similar significance for Theme 3 severity while leaving Theme 3 recovery imprecise. Using the maximum deficit over days 3–7 (rather than day 3 alone) as the severity outcome produced larger Theme 3 (+0.56 nW/cm^2^/sr; *P* < 0.001) and Theme 4 (+0.27; *P* < 0.01) associations.

### Land cover sensitivity:

To test whether vegetation and urbanisation differences confound the SVI-outage associations, we re-estimated all three stages with tract-level land cover controls. Tree canopy cover (MODIS VCF) was available for 99.6% of the analytic sample; impervious surface cover (NLCD) was available for the same tracts (109,708 observations). Tree canopy was negatively correlated with SVI Theme 3 (*r* = −0.38) and impervious surface positively correlated (*r* = +0.41); the two land cover variables were strongly inversely correlated (*r* = −0.72), jointly capturing the urbanisation gradient from dense cores (high impervious, greater infrastructure redundancy) to dispersed neighbourhoods (high canopy, potentially more vulnerable distribution networks). We report models with tree canopy alone, impervious surface alone, and both together, as well as an intentionally stringent specification combining within-county × hurricane fixed effects with both land cover controls. Because land cover is plausibly on the causal pathway from segregation to infrastructure quality, attenuation under this specification may reflect confounding or mediation (see Discussion). Results are reported in Extended Data Table 6.

### Ecological inference:

All analyses use census-tract-level exposures and outcomes. A tract with high minority status percentile contains residents of all racial and ethnic backgrounds who share the same electrical infrastructure; this ecological design is appropriate for studying infrastructure investment decisions but does not support individual-level causal inference.

## Extended Data

**Extended Data Fig. 1 |:**
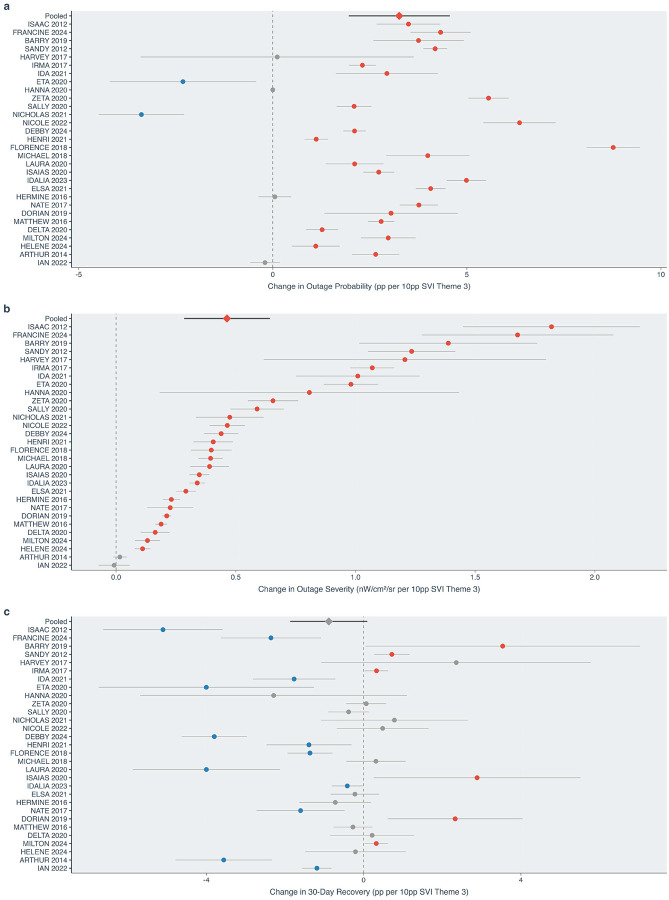
Hurricane-specific Theme 3 associations across three outage stages. **a**, Outage probability (pp per 10pp SVI). **b**, Outage severity (nW/cm^2^/sr per 10pp SVI). **c**, 30-day recovery rate (pp per 10pp SVI; negative = slower). Each point shows a hurricane-specific SVI Theme 3 (Minority Status/Language) coefficient from a single-theme model with wind speed and distance controls and heteroskedasticity-robust standard errors. Hurricanes ordered by severity estimate (panel b). Red, positive and significant (*P* < 0.05); blue, negative and significant; grey, not significant. Diamonds show pooled fixed-effects estimates with *t*(16) CIs from two-way clustering (state + hurricane).

**Extended Data Fig. 2 |:**
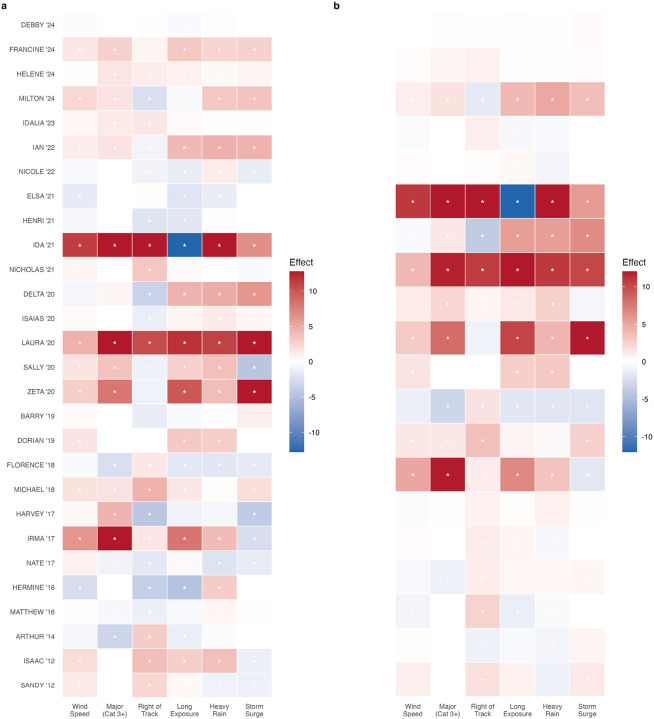
Storm characteristic effects on hurricane-related outages by hurricane. **a,** Effects on outage severity. **b**, Effects on 30-day recovery rate. Storm characteristics include: wind speed (standardised within hurricane), major hurricane (Category 3+), right of track (dangerous semicircle), long exposure (above 75th percentile duration), heavy rain (above median precipitation) and storm surge (surge exposure). Red indicates the characteristic worsens outcomes; blue indicates it improves outcomes. Asterisks indicate *P* < 0.05. White cells indicate the variable could not be estimated due to lack of variation or collinearity within that hurricane.

**Extended Data Table 1.** Three-stage association between SVI themes and hurricane-related outage outcomes. All effects per 10-percentile-point increase in SVI theme. Stage 1 (outage probability) and Stage 2 (unconditional outage severity) are estimated on all 156,032 analytic tract-hurricane observations. Stage 3 (recovery pace) is estimated on 50,441 tract-hurricane observations with detectable outages, controlling for initial deficit magnitude. Negative recovery coefficients indicate slower recovery.

SVI Theme	Stage 1: Outage Probability (pp)	Stage 2: Severity (nW/cm^2^/sr)	Stage 3: Recovery (pp)
Socioeconomic	−0.84 (−1.17, −0.50)***	+0.020 (−0.053, +0.093)	−0.29 (−0.57, −0.01)*
Household Composition	−0.41 (−0.88, +0.05)	−0.253 (−0.393, −0.113)**	+0.39 (−0.04, +0.81)
Minority Status	+3.47 (+2.44, +4.50)***	+0.467 (+0.297, +0.637)***	−0.48 (−1.09, +0.14)
Housing Type	+0.72 (+0.44, +1.00)***	+0.229 (+0.108, +0.351)***	−0.42 (−0.67, −0.16)**

Significance: * *P* < 0.05, ** *P* < 0.01, *** *P* < 0.001; 95% CIs use two-way clustered standard errors (state + hurricane).

**Extended Data Table 2.** Effect modification of social vulnerability associations by storm characteristics. Four interaction models show how heavy rainfall (above sample median precipitation), long exposure (above the 75th percentile duration), right-of-track position, and wind speed (standardised within hurricane) modify the Theme 3 (Minority Status/Language) association at each stage. Models use Theme 3 only (not the four-theme joint specification) to avoid overparameterisation. Interaction coefficients represent the additional effect per 10-percentile-point increase in SVI Theme 3 when the storm characteristic is present. Probability and recovery interaction coefficients are reported in percentage points. Heavy rainfall, long exposure, right-of-track position, and wind speed each increased the Theme 3 severity association, with heavy rainfall showing a +0.49 nW/cm^2^/sr interaction (*P* < 0.05). Two-way clustered standard errors (state + hurricane).

Modifier	P(Outage) (pp)	Severity (nW)	Recovery (pp)
Theme 3 × Heavy Rain	−0.20 (0.83)	+0.49 (0.18)*	+1.04 (0.58)
Theme 3 × Long Exposure	−0.58 (1.01)	+0.47 (0.15)**	+0.66 (0.39)
Theme 3 × Right of Track	+1.08 (0.67)	+0.50 (0.14)**	+0.23 (0.14)
Theme 3 × Wind (std)	−0.76 (0.63)	+0.23 (0.07)**	−0.16 (0.36)

Significance: * *P* < 0.05, ** *P* < 0.01, *** *P* < 0.001.

**Extended Data Table 3.** Deficit threshold sensitivity analysis. Coefficients (s.e.) from the four-theme joint model estimated at five deficit inclusion thresholds. **All SVI coefficients are on the original 0–1 scale; divide by 10 for per-10-percentile-point equivalents used in main text.** Two-way clustered s.e. (state + hurricane).

Theme	None	>0.5 nW	>1 nW	>2 nW	>3 nW
Theme 1 (SES)	0.03 (1.09)	0.99 (0.45)*	1.33 (0.51)*	1.71 (0.6)*	1.73 (0.63)*
Theme 2 (Household)	−0.06 (0.62)	−2.29 (0.35)***	−2.4 (0.38)***	−2.67 (0.45)***	−2.9 (0.46)***
Theme 3 (Minority)	1.01 (0.44)*	3.64 (0.81)***	3.46 (1.12)**	3.63 (1.56)*	3.83 (1.94)†
Theme 4 (Housing)	1.39 (0.14)***	3.51 (0.78)***	3.71 (0.75)***	4.14 (0.78)***	4.66 (0.84)***

Significance: † *P* < 0.1, * *P* < 0.05, ** *P* < 0.01, *** *P* < 0.001.

**Extended Data Table 4.** Wild cluster bootstrap validation of severity models. Analytical estimates with two-way clustered standard errors and wild cluster bootstrap confidence intervals are shown for the main SVI themes, selected SVI components, and selected storm-characteristic models. **All SVI Theme estimates are on the original 0–1 scale; divide by 10 to convert to the per-10-percentile-point scale used in the main text.** SVI component and storm-characteristic estimates are already reported per 10 percentile points. Bootstrap intervals are used here as a precision check for the severity models and should be interpreted alongside the main two-way clustered estimates.

Category	Variable	Estimate	Two-Way SE	Two-Way p	Boot 95% CI	CI Excludes 0
SVI Theme (0–1 scale)	Theme 1: Socioeconomic	0.97	1.03	0.361	(−0.64, 2.68)	No
SVI Theme (0–1 scale)	Theme 2: Household	−5.58	1.21	<0.001	(−7.70, −3.77)	Yes
SVI Theme (0–1 scale)	Theme 3: Minority	5.45	1.14	<0.001	(3.20, 7.32)	Yes
SVI Theme (0–1 scale)	Theme 4: Housing	5.39	1.08	<0.001	(3.25, 7.47)	Yes
SVI Component (per 10pp)	Minority	0.50	0.15	0.003	(0.28, 0.75)	Yes
SVI Component (per 10pp)	Limited English	2.16	0.50	<0.001	(0.80, 3.46)	Yes
SVI Component (per 10pp)	Below 150% Poverty	0.87	0.18	<0.001	(0.58, 1.25)	Yes
SVI Component (per 10pp)	Unemployed	0.74	0.18	<0.001	(0.34, 1.18)	Yes
SVI Component (per 10pp)	Housing Cost Burden	1.55	0.29	<0.001	(1.05, 2.09)	Yes
SVI Component (per 10pp)	Age 65+	−0.51	0.08	<0.001	(−0.77, −0.26)	Yes
SVI Component (per 10pp)	Single Parent	0.09	0.40	0.828	(−0.38, 0.60)	No
SVI Component (per 10pp)	Multi-unit Housing	1.43	0.25	<0.001	(1.13, 1.81)	Yes
SVI Component (per 10pp)	Group Quarters	1.06	0.19	<0.001	(0.74, 1.46)	Yes
Storm Characteristic	Wind × Hours (std)	3.44	0.71	<0.001	(1.60, 5.02)	Yes
Storm Characteristic	Major Hurricane (Cat 3+)	4.67	1.50	0.006	(0.39, 9.71)	Yes
Storm Characteristic	Long Exposure	3.76	0.92	<0.001	(1.76, 6.11)	Yes
Storm Characteristic	Heavy Rainfall	2.52	1.26	0.061	(0.79, 4.72)	Yes
Storm Characteristic	Rural Area	−5.38	1.57	0.003	(−7.46, −3.82)	Yes

**Extended Data Table 5.** Robustness of severity associations across alternative specifications. **Panel A**: Theme 3 and Theme 4 severity coefficients (nW/cm^2^/sr per 10pp SVI increase) from the four-theme joint model under five specifications. The main model uses hurricane fixed effects. ‘Relative deficit’ uses deficit/baseline as the outcome; ‘baseline control’ adds baseline radiance as a covariate. ‘Within-county FE’ uses county × hurricane fixed effects, comparing tracts within the same county during the same hurricane to absorb hazard geography differences. ‘2017–2024 subsample’ restricts to storms closer to the 2020 SVI vintage. Baseline radiance is endogenous to infrastructure quality (see Discussion), so the within-county specification provides a more relevant geographic robustness check. **Panel B**: Missingness characterisation comparing tracts with observed versus missing post-storm radiance. Missingness is concentrated in high-rainfall, high-radiance tracts. **Panel C**: Three-stage results in the 2017–2024 subsample, which is better aligned with the 2020 SVI vintage. Theme 3 probability and severity remain similar to the full-sample estimates; Theme 3 recovery remains negative but imprecise. **Panel D**: Utility-territory sensitivity. Public utility service-territory polygons were linked to 2020 Census tract geometries, assigning 35,751 of 39,467 unique tracts (90.6%) to 327 utilities and covering 96.6% of Stage 1–2 observations and 98.0% of recovery observations. Theme 3 probability and severity associations persist under utility fixed effects and utility × hurricane fixed effects, whereas Theme 3 recovery remains negative but imprecise. Two-way clustered standard errors (state + hurricane in Panels A–C; utility + hurricane in Panel D).

Panel A: Severity estimates across specifications				
Specification	Theme 3 Severity (nW)	Theme 4 Severity (nW)		
Main model (hurricane FE)	0.467***	0.229***		
Relative deficit (deficit/baseline)	−0.002	0.001		
Absolute deficit + baseline control	−0.241**	−0.031		
Within-county × hurricane FE	0.142**	0.250***		
2017–2024 subsample	0.469***	0.246**		
Panel B: Missingness characterisation				
Variable	Observed	Missing	Difference	P
SVI Theme 1	0.56	0.54	−0.02	<0.001
SVI Theme 2	0.52	0.50	−0.01	<0.001
SVI Theme 3	0.54	0.58	+0.04	<0.001
SVI Theme 4	0.48	0.51	+0.02	<0.001
Wind speed (kt)	65.07	57.73	−7.35	<0.001
Baseline radiance (nW)	20.13	29.20	+9.07	<0.001

Panel A: Severity estimates across specifications				
Specification	Theme 3 Severity (nW)	Theme 4 Severity (nW)		
Precipitation (mm)	74.82	106.85	+32.03	<0.001
Panel C: 2017--2024 temporal mismatch sensitivity				
Stage	Theme 3	Theme 4		
Probability (pp)	+3.44***	+0.69***		
Severity (nW/cm^2^/sr)	+0.47***	+0.25**		
Recovery (pp)	−0.36	0.41***		
Panel D: Utility-territory sensitivity				
Contrast	Matched sample	Utility FE	Utility × hurricane FE	
Probability - Theme 3	+3.39***	+3.37***	+3.39***	
Probability - Theme 4	+0.77***	+0.66***	+0.66***	
Severity - Theme 3	+0.46***	+0.47***	+0.46***	
Severity - Theme 4	+0.24***	+0.22**	+0.22**	
Recovery - Theme 3	−0.45	−0.69	−0.57	
Recovery - Theme 4	−0.39**	−0.42***	−0.42***	

Significance: * *P* < 0.05, ** *P* < 0.01, *** *P* < 0.001

**Extended Data Table 6.** Land cover and storm intensity sensitivity analyses. Panel A: Theme 3 and Theme 4 coefficients from the four-theme joint model with hurricane fixed effects under four land-cover specifications: base (no land cover controls), + tree canopy only (MODIS VCF), + impervious surface only (NLCD), and + both. Tree canopy and impervious surface are strongly inversely correlated (*r* = −0.72), jointly capturing the urbanisation gradient. Theme 3 severity attenuates sharply with land-cover adjustment, whereas Theme 4 severity remains positive. **Panel B**: Within-county × hurricane fixed effects combined with land-cover controls. This intentionally stringent specification removes both geographic sorting and much of the built-environment pathway; attenuation to zero is therefore expected if land cover mediates the association. **Panel C:** Storm intensity and baseline-radiance stratification. Theme 3 coefficients (0–1 SVI scale; divide by 10 for per-10pp equivalents) from single-theme models with hurricane fixed effects are shown by hurricane category (Cat 1–2 versus Cat 3+) and baseline radiance (above/below median). Two-way clustered standard errors (state + hurricane).

Panel A: Hurricane fixed effects with land cover controls						
Stage	Theme	1. Base	2. + Tree	3. + Impervious	4. + Both	Δ
Probability	3	+3.500***	+ 1.659***	+0.807***	+0.704***	80%
Probability	4	+0.696***	+0.368*	+0.110	+0.108	85%
Severity	3	+0.466***	+0.213*	−0.026	−0.014	103%
Severity	4	+0.213***	+0.168**	+0.106*	+0.107*	50%
Recovery	3	−0.485	−0.417	−0.271	−0.312	36%
Recovery	4	−0.399**	−0.293**	−0.248*	−0.239*	40%
Panel B: Within-county × hurricane FE ± land cover controls						
Stage	Theme	Within-county FE	+ Land cover			
Probability	3	+ 1.957***	+ 1.164***			
Probability	4	+0.773***	+0.268**			
Severity	3	+0.078***	−0.013			
Severity	4	+0.120***	+0.059**			
Recovery	3	−0.284**	−0.245**			
Recovery	4	−0.232*	−0.160			
Panel C: Theme 3 stratification by storm intensity and baseline radiance						
Stage	Stratification	Estimate (SE)	N			
Occurrence	Cat 1–2	+0.065 (0.063)	125,717			

Panel A: Hurricane fixed effects with land cover controls						
Stage	Theme	1. Base	2. + Tree	3. + Impervious	4. + Both	Δ
Occurrence	Cat 3+	+0.017 (0.025)	38,590			
Occurrence	Low baseline radiance	+0.100 (0.054)	84,452			
Occurrence	High baseline radiance	−0.045 (0.026)	79,855			
Severity	Cat 1–2	+6.109 (0.838)***	68,056			
Severity	Cat 3+	+5.431 (1.331)**	22,371			
Recovery	Cat 1–2	−3.426 (5.657)	67,227			
Recovery	Cat 3+	−12.207 (4.758)*	22,334			

Significance: * *P* < 0.05, ** *P* < 0.01, *** *P* < 0.001.

**Extended Data Table 7.** SVI component decomposition for outage severity and recovery. Each row shows the coefficient from a separate single-variable model (one SVI component per model) with hurricane fixed effects, wind speed and distance-to-track controls, and two-way clustered standard errors (state + hurricane). **Panel A:** Severity (nW/cm^2^/sr per 10-percentile-point increase in the component variable). **Panel B**: Recovery (percentage points per 10-percentile-point increase; negative = slower recovery). Components are grouped by SVI theme. Because components within each theme are correlated, estimates are from separate models and are not additive.

Panel A: Severity (nW/cm^2^/sr per 10pp)			
Theme	Component	Estimate (SE)	P
Socioeconomic	Below 150% poverty	+0.49 (0.11)	<0.001
Socioeconomic	Unemployed	+0.59 (0.15)	0.001
Socioeconomic	Housing cost burden	+1.25 (0.24)	<0.001
Socioeconomic	No HS diploma	+0.34 (0.22)	0.148
Socioeconomic	Uninsured	+0.83 (0.23)	0.002
Household	Age 65+	−0.46 (0.11)	<0.001
Household	Age 17 or younger	−0.76 (0.27)	0.013
Household	Disability	−0.71 (0.16)	<0.001
Household	Single parent	+0.63 (0.16)	<0.001
Minority	Minority	+0.45 (0.08)	<0.001
Minority	Limited English	+1.87 (0.28)	<0.001
Housing	Multi-unit housing	+1.00 (0.20)	<0.001
Housing	Mobile homes	−0.77 (0.23)	0.004
Housing	Crowded housing	+1.72 (0.52)	0.004
Housing	No vehicle	+1.84 (0.36)	<0.001
Housing	Group quarters	+0.34 (0.10)	0.003
Panel B: Recovery (pp per 10pp)			
Theme	Component	Estimate (SE)	P
Socioeconomic	Below 150% poverty	−0.01 (0.00)	<0.001
Socioeconomic	Unemployed	−0.01 (0.00)	0.135
Socioeconomic	Housing cost burden	−0.02 (0.00)	<0.001
Socioeconomic	No HS diploma	−0.01 (0.00)	0.037
Panel A: Severity (nW/cm^2^/sr per 10pp)			
Theme	Component	Estimate (SE)	P
Socioeconomic	Uninsured	−0.02 (0.01)	0.006
Household	Age 65+	−0.00 (0.00)	0.722
Household	Age 17 or younger	+0.02 (0.01)	0.011
Household	Disability	−0.00 (0.00)	0.329
Household	Single parent	−0.00 (0.01)	0.609
Minority	Minority	−0.01 (0.00)	0.017
Minority	Limited English	−0.03 (0.01)	<0.001
Housing	Multi-unit housing	−0.02 (0.00)	<0.001
Housing	Mobile homes	+0.01 (0.00)	0.013
Housing	Crowded housing	−0.03 (0.01)	0.003
Housing	No vehicle	−0.02 (0.00)	<0.001
Housing	Group quarters	−0.01 (0.00)	0.080

Significance: * *P* < 0.05, ** *P* < 0.01, *** *P* < 0.001.

## Figures and Tables

**Figure 1:**
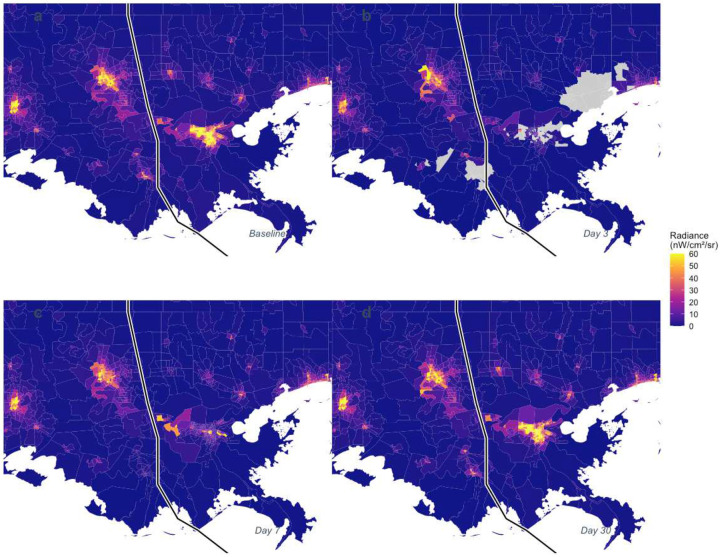
Satellite detection of hurricane-related outages. VIIRS nighttime radiance before and after Hurricane Ida (2021, Louisiana and Mississippi). a, Baseline radiance 15–30 days before landfall. b, Day 3 post-landfall showing peak outage. c, Day 7 showing partial recovery. d, Day 30 showing near-complete recovery. White line, hurricane track; star, landfall location. Grey tracts, missing satellite data due to cloud cover.

**Table 1:** Hurricane characteristics (2012–2024). The States column lists states containing at least one census tract whose centroid fell within the exposure definition at any point during the storm. Because exposure was defined using broad storm wind radii (R34, 34-knot threshold), some listed states may appear counterintuitive relative to the landfall state alone. SVI columns show the mean tract-level percentile among exposed tracts for each theme (0–1 scale, where 1 = highest vulnerability). Regression effects in main text are reported per 10-percentile-point (0.10-unit) increase. Wind speed is mean tract-level maximum sustained wind (knots) across exposed observations. Four hurricanes excluded (see Methods).

Hurricane	Landfall	States	Tracts	SES (T1)	HH/Disab (T2)	Minority (T3)	Housing (T4)	Wind
Zeta (2020)	Oct 29, 2020	AL, LA, MS	3,699	0.60	0.53	0.53	0.48	83
Sandy (2012)	Oct 29, 2012	CT, DE, MD, NJ, NY, PA, VA	3,942	0.43	0.45	0.44	0.46	68
Sally (2020)	Sep 16, 2020	AL, FL, MS	1,827	0.59	0.52	0.49	0.47	58
Nicole (2022)	Nov 10, 2022	FL	5,028	0.58	0.55	0.56	0.49	56
Nicholas (2021)	Sep 14, 2021	TX	3,938	0.62	0.59	0.71	0.50	50
Nate (2017)	Oct 08, 2017	AL, LA, MS	2,512	0.59	0.52	0.52	0.46	57
Milton (2024)	Oct 10, 2024	FL	4,823	0.58	0.55	0.56	0.49	76
Michael (2018)	Oct 10, 2018	AL, FL, GA	8,307	0.57	0.51	0.53	0.46	98
Matthew (2016)	Oct 08, 2016	FL, GA, NC, SC	10,027	0.58	0.53	0.55	0.49	96
Laura (2020)	Aug 27, 2020	LA, TX	3,468	0.61	0.56	0.62	0.49	78
Isaias (2020)	Aug 04, 2020	MD, NC, NJ, NY, VA	9,020	0.45	0.46	0.46	0.46	60
Isaac (2012)	Aug 29, 2012	LA, MS	1,805	0.61	0.53	0.56	0.51	52
Irma (2017)	Sep 11, 2017	FL, GA, SC	8,600	0.58	0.53	0.56	0.48	59
Ida (2021)	Aug 29, 2021	LA, MS	1,941	0.62	0.54	0.55	0.51	58
Idalia (2023)	Aug 30, 2023	FL, GA	6,773	0.57	0.51	0.54	0.45	95
Ian (2022)	Sep 28, 2022	FL, NC, SC	6,447	0.58	0.55	0.53	0.50	89
Hermine (2016)	Sep 02, 2016	FL, GA, NC, SC	8,351	0.56	0.50	0.52	0.47	51
Henri (2021)	Aug 22, 2021	CT, MA, NJ, NY, RI	9,281	0.46	0.48	0.54	0.54	19
Helene (2024)	Sep 27, 2024	FL, GA, NC, SC, TN, VA	13,291	0.56	0.51	0.52	0.48	66
Harvey (2017)	Aug 26, 2017	TX	1,103	0.70	0.70	0.79	0.54	55
Hanna (2020)	Jul 26, 2020	TX	209	0.68	0.66	0.78	0.57	46
Francine (2024)	Sep 11, 2024	LA	1,038	0.59	0.52	0.54	0.49	68
Florence (2018)	Sep 14, 2018	NC, SC	903	0.56	0.54	0.41	0.52	33
Eta (2020)	Nov 12, 2020	FL	4,815	0.58	0.54	0.55	0.48	58
Elsa (2021)	Jul 07, 2021	FL, GA, NC, SC	8,156	0.57	0.53	0.53	0.49	30
Dorian (2019)	Sep 06, 2019	FL, GA, NC, SC	11,609	0.57	0.52	0.55	0.48	104
Delta (2020)	Oct 10, 2020	LA	1,334	0.60	0.52	0.55	0.50	68
Debby (2024)	Aug 05, 2024	FL, GA, NC, SC	9,183	0.57	0.52	0.55	0.47	19
Barry (2019)	Jul 13, 2019	LA	632	0.58	0.53	0.51	0.52	43
Arthur (2014)	Jul 04, 2014	NC, SC, VA	6,138	0.51	0.48	0.51	0.47	68

## Data Availability

All source datasets used in this study are publicly available. VIIRS Black Marble data are available from NASA LAADS DAAC (https://ladsweb.modaps.eosdis.nasa.gov/). CDC Social Vulnerability Index data are available from ATSDR (https://www.atsdr.cdc.gov/placeandhealth/svi/). IBTrACS hurricane track data are available from NOAA NCEI (https://www.ncei.noaa.gov/products/international-best-track-archive). PRISM daily temperature data are available from Oregon State University (https://prism.oregonstate.edu/). ERA5-Land reanalysis precipitation data are available from ECMWF (https://cds.climate.copernicus.eu/). MODIS Vegetation Continuous Fields (MOD44B) tree canopy data are available from NASA LP DAAC (https://lpdaac.usgs.gov/products/mod44bv006/). NLCD impervious surface data are available from USGS (https://www.mrlc.gov/). The processed tract-level analysis dataset used for the reported models is included in the project repository associated with this manuscript and will be deposited in a public archive upon publication.

## Code availability:

All analysis code used to generate the reported results, including Google Earth Engine scripts for data extraction and R scripts for statistical analyses and figure generation, is included in the project repository associated with this manuscript and will be deposited in a public archive upon publication.

## References

[1] Kossin J. P., Knapp K. R., Olander T. L. & Velden C. S. Global increase in major tropical cyclone exceedance probability over the past four decades. Proceedings of the National Academy of Sciences 117, 11975–11980 (2020). 10.1073/pnas.1920849117 PMC7275711 32424081

[2] Emanuel K. Assessing the present and future probability of Hurricane Harvey’s rainfall. Proceedings of the National Academy of Sciences 114, 12681–12684 (2017). 10.1073/pnas.1716222114 PMC5715789 29133388

[3] Jiang L. Characterizing global tropical cyclone events of 2024. Environmental Research Letters https://doi.org/10.1088/1748-9326/ae34cc (2025) doi:10.1088/1748-9326/ae34cc.

[4] Anderson G. B. & Bell M. L. Lights out: Impact of the August 2003 power outage on mortality in New York, NY. Epidemiology 23, 189–193 (2012). 22252408 10.1097/EDE.0b013e318245c61c PMC3276729

[5] Casey J. A., Fukurai M., Hernandez D., Power outages and community health: A narrative review. Current Environmental Health Reports 7, 371–383 (2020). 33179170 10.1007/s40572-020-00295-0 PMC7749027

[6] Parks R. M. Tropical cyclone exposure is associated with increased hospitalization rates in older adults. Nature Communications 12, 1545 (2021). 10.1038/s41467-021-21777-1 PMC7943804 33750775

[7] Parks R. M. Association of tropical cyclones with county-level mortality in the US. JAMA 327, 946–955 (2022). 35258534 10.1001/jama.2022.1682 PMC8905400

[8] U.S. Energy Information Administration. Hurricane Irma caused the largest power outage in Florida’s history. (2017).

[9] U.S. Congress. Infrastructure investment and jobs act. (2021).

[10] Biden J. R. Executive order 14008: Tackling the climate crisis at home and abroad. Federal Register vol. 86 7619–7633 (2021).

[11] Trump D. J. Executive order 14148: Initial rescissions of harmful executive orders and actions. Federal Register vol. 90 8237 (2025).

[12] Florida Legislature. Public utility storm protection plans. (2019).

[13] Brelsford C. A dataset of recorded electricity outages by United States county 2014–2022. Scientific Data 11, 271 (2024). 38443375 10.1038/s41597-024-03095-5 PMC10915145

[14] Eto J. H. & Hamachi LaCommare K. A quantitative assessment of utility reporting practices for reporting electric power distribution events. in 2012 IEEE power and energy society general meeting 1–6 (San Diego, CA, 2012). doi:10.1109/PESGM.2012.6344626.

[15] Ganz S. C., Duan C. & Ji C. Socioeconomic vulnerability and differential impact of severe weather-induced power outages. PNAS Nexus 2, pgad295 (2023). 37795271 10.1093/pnasnexus/pgad295 PMC10547019

[16] Mitsova D., Esnard A.-M., Sapat A. & Lai B. S. Socioeconomic vulnerability and electric power restoration timelines in Florida: The case of Hurricane Irma. Natural Hazards 94, 689–709 (2018).

[17] Coleman N., Esmalian A. & Mostafavi A. Equitable resilience in infrastructure systems: Empirical assessment of disparities in hardship experiences of vulnerable populations during service disruptions. Natural Hazards Review 21, (2020).

[18] Dominianni C., Lane K., Johnson S., Health impacts of citywide and localized power outages in New York City. Environmental Health Perspectives 126, 067003 (2018). 29894117 10.1289/EHP2154 PMC6084843

[19] An B. Y. & Kim J. High-resolution analysis of power outages and extreme weather events exposes environmental injustice in electric grid restoration. (2025) doi:10.21203/rs.3.rs-7878685/v1.

[20] Switzer D. & Teodoro M. P. The color of drinking water: Class, race, ethnicity, and Safe Drinking Water Act compliance. Journal AWWA 109, 40–45 (2017).

[21] Karner A. & Golub A. Comparison of two common approaches to public transit service equity evaluation. Transportation Research Record 2531, 170–179 (2016).

[22] Brockway A. M., Conde J. & Callaway D. Inequitable access to distributed energy resources due to grid infrastructure limits in California. Nature Energy 6, 892–903 (2021).

[23] Elvidge C. D., Baugh K. E., Zhizhin M. & Hsu F.-C. Why VIIRS data are superior to DMSP for mapping nighttime lights. Proceedings of the Asia-Pacific Advanced Network 35, 62–69 (2013).

[24] Bennett M. M. & Smith L. C. Advances in using multitemporal night-time lights satellite imagery to detect, estimate, and monitor socioeconomic dynamics. Remote Sensing of Environment 192, 176–197 (2017).

[25] Cao C., Shao X. & Uprety S. Detecting light outages after severe storms using the S-NPP/VIIRS day/night band radiances. EEE Geoscience and Remote Sensing Letters 10, 1582–1586 (2013).

[26] Flanagan B. E., Gregory E. W., Hallisey E. J., Heitgerd J. L. & Lewis B. A social vulnerability index for disaster management. Journal of Homeland Security and Emergency Management 8, Article 3 (2011).

[27] CDC/ATSDR. CDC/ATSDR Social Vulnerability Index 2020 documentation. (2022).

[28] Rothstein R. The Color of Law: A Forgotten History of How Our Government Segregated America. (Liveright Publishing, 2017).

[29] Pulido L. Rethinking environmental racism: White privilege and urban development in Southern California. Annals of the Association of American Geographers 90, 12–40 (2000).

[30] Collins T. W., Grineski S. E., Chakraborty J. & Flores A. B. Environmental injustice and Hurricane Harvey: A household-level study of socially disparate flood exposures in Greater Houston, Texas, USA. Environmental Research 179, 108772 (2019). 31593835 10.1016/j.envres.2019.108772

[31] Howell J. & Elliott J. R. As disaster costs rise, so does inequality. Socius 4, 1–3 (2018).

[32] Jenkins K., McCauley D., Heffron R., Stephan H. & Rehner R. Energy justice: A conceptual review. Energy Research & Social Science 11, 174–182 (2016).

[33] Bouchama A. & Knochel J. P. Heat stroke. New England Journal of Medicine 346, 1978–1988 (2002). 12075060 10.1056/NEJMra011089

[34] Semenza J. C. Heat-related deaths during the July 1995 heat wave in Chicago. New England Journal of Medicine 335, 84–90 (1996). 8649494 10.1056/NEJM199607113350203

[35] Parks R. M. Short-term excess mortality following tropical cyclones in the United States. Science Advances 9, eadg6633 (2023). 37585525 10.1126/sciadv.adg6633 PMC10431701

[36] Spriggs R. Tropical cyclone exposure and psychoactive drug-related death rates. JAMA Network Open 9, e2560183 (2026). 41719042 10.1001/jamanetworkopen.2025.60183 PMC12924108

[37] Hernández D. Understanding ’energy insecurity’ and why it matters to health. Social Science & Medicine 167, 1–10 (2016). 27592003 10.1016/j.socscimed.2016.08.029 PMC5114037

[38] Bednar D. J. & Reames T. G. Recognition of and response to energy poverty in the United States. Nature Energy 5, 432–439 (2020).

[39] Kishore N. Mortality in Puerto Rico after Hurricane Maria. New England Journal of Medicine 379, 162–170 (2018). 29809109 10.1056/NEJMsa1803972

[40] Knutson T. Tropical cyclones and climate change assessment: Part II: Projected response to anthropogenic warming. Bulletin of the American Meteorological Society 101, E303–E322 (2020).

[41] Knapp K. R., Kruk M. C., Levinson D. H., Diamond H. J. & Neumann C. J. The International Best Track Archive for Climate Stewardship (IBTrACS). Bulletin of the American Meteorological Society 91, 363–376 (2010).

[42] Willoughby H. E., Darling R. W. & Rahn M. E. Parametric representation of the primary hurricane vortex. Part II: A new family of sectionally continuous profiles. Monthly Weather Review 134, 1102–1120 (2006).

[43] Roman M. O., Wang Z., Sun Q., NASA’s Black Marble nighttime lights product suite. Remote Sensing of Environment 210, 113–143 (2018).

[44] Gorelick N., Hancher M., Dixon M., Google Earth Engine: Planetary-scale geospatial analysis for everyone. Remote Sensing of Environment 202, 18–27 (2017).

[45] Daly C. Physiographically sensitive mapping of climatological temperature and precipitation across the conterminous United States. International Journal of Climatology 28, 2031–2064 (2008).

[46] Bergé L. Efficient estimation of maximum likelihood models with multiple fixed-effects: The R package FENmlm. CREA Discussion Papers 13, (2018).

